# A comparison of the CAM-ICU and the NEECHAM Confusion Scale in intensive care delirium assessment: an observational study in non-intubated patients

**DOI:** 10.1186/cc6790

**Published:** 2008-02-18

**Authors:** Bart Van Rompaey, Marieke J Schuurmans, Lillie M Shortridge-Baggett, Steven Truijen, Monique Elseviers, Leo Bossaert

**Affiliations:** 1 University of Antwerp, Faculty of Medicine, Division of Nursing Science and Midwifery, Belgium, Universiteitsplein 1, 2610 Wilrijk, Belgium; 2 University College of Antwerp, Department of Health Sciences, J. De Boeckstraat 10, 2170 Merksem, Belgium; 3 University of Professional Education Utrecht, Department of Healthcare, Bolognalaan 101, postbus 85182, 3508 AD Utrecht, The Netherlands; 4 Pace University, Lienhard School of Nursing, Lienhard Hall, Pleasantville, NY 10570, USA; 5 University Hospital of Antwerp, Intensive Care Department, Belgium, University of Antwerp, and Faculty of Medicine, Belgium, Universiteitsplein 1, 2610 Wilrijk, Belgium

## Abstract

**Background:**

Several reports indicate a high incidence of intensive care delirium. To develop strategies to prevent this complication, validated instruments are needed. The Confusion Assessment Method for the Intensive Care Unit (CAM-ICU) is widely used. A binary result diagnoses delirium. The Neelon and Champagne (NEECHAM) Confusion Scale recently has been validated for use in the ICU and has a numeric assessment. This scale allows the patients to be classified in four categories: non-delirious, at risk, confused, and delirious. In this study, we investigated the results of the NEECHAM scale in comparison with the CAM-ICU.

**Methods:**

A consecutive sample of 172 non-intubated patients in a mixed ICU was assessed after a stay in the ICU for at least 24 hours. All adult patients with a Glasgow Coma Scale score of greater than 9 were included. A nurse researcher simultaneously assessed both scales once daily in the morning. A total of 599 paired observations were made.

**Results:**

The CAM-ICU showed a 19.8% incidence of delirium. The NEECHAM scale detected incidence rates of 20.3% for delirious, 24.4% for confused, 29.7% for at risk, and 25.6% for normal patients. The majority of the positive CAM-ICU patients were detected by the NEECHAM scale. The sensitivity of the NEECHAM scale was 87% and the specificity was 95%. The positive predictive value and the negative predictive value were 79% and 97%, respectively. The diagnostic capability in cardiac surgery patients proved to be lower than in other patients.

**Conclusion:**

In non-intubated patients, the NEECHAM scale identified most cases of delirium which were detected by the CAM-ICU. Additional confused patients were identified in the categorical approach of the scale. The NEECHAM scale proved to be a valuable screening tool compared with the CAM-ICU in the early detection of intensive care delirium by nurses.

## Introduction

Delirium is a well-known acute syndrome in the intensive care unit (ICU). A physical cause induces a fluctuating disturbance of the cognitive processes in the brain. The patient encounters periods of inattention in combination with disorganized thinking or a changed level in consciousness. The process is observed as a hypoactive, hyperactive, or mixed type. The hyperactive type is the least frequent one although it is the easiest to detect [1,2]. Incidence rates of intensive care delirium were reported in a range from 11% to 87% [3,4]. To develop strategies to prevent or cure this complication, validated instruments for diagnosing, screening, and quantifying are needed.

The standard assessment of delirium is performed when a psychiatrist uses the *Diagnostic and Statistical Manual of Mental Disorders *(DSM) criteria [5]. The development of internationally accepted diagnostic tools created the opportunity to compare and verify the onset and process of intensive care delirium without the need for consulting a psychiatrist. The Confusion Assessment Method (CAM) [6,7] is a well-validated and frequently used tool. The scale was designed to be used by non-psychiatric physicians and trained researchers. Because the patient in intensive care is not always able to communicate verbally, the CAM was adapted for screening intubated or artificially ventilated patients. The Confusion Assessment Method for the Intensive Care Unit (CAM-ICU) [8] is widely accepted as the standard in intensive care delirium assessment. This assessment tool was based on the DSM-IV criteria and diagnoses the delirious state by a yes or no answer to a four-point algorithm (Appendix 1). A positive answer to this algorithm indicates delirium and a negative answer indicates a normal cognitive state. Nevertheless, the results of this scale are limited by its binomial approach of the evaluation of delirium and the fact that it is a one-point-in-time assessment.

The Neelon and Champagne (NEECHAM) Confusion Scale [9] was developed a few years later based on daily nursing practice. In this scale, the nurses' 24-hour assessment of the level of processing information, the level of behavior, and the physiological condition rate the patient on a 30 to 0 scale classifying him or her in one of four categories (Appendix 2). The cutoff values of 30 to 27 for 'non-delirious' (normal), 26 or 25 for 'at risk', and 24 to 20 for 'early to mild confusion' (mild confusion) were standardized. Validation for delirium against the DSM-III-R criteria was performed for the scores 19 to 0 ('moderate to severe confusion') in the original development of the scale. Consequently, the delirious state can be assessed and changes in the cognitive function of the patient can be monitored. The NEECHAM scale is reliable for the detection of delirium by nurses in the general hospital population [10,11] and recently has been validated for use in the intensive care environment [12,13]. In this study, we investigated the NEECHAM scale in comparison with the CAM-ICU in a non-intubated intensive care population.

## Materials and methods

All patients were admitted to the intensive care department of the Antwerp University Hospital (625 beds). The department has a capacity of 39 beds and admits more than 2,000 patients each year. This department is divided in five units of seven or nine beds. These units are preferentially, but not exclusively, specialized in treating cardiosurgical, surgical, or medical intensive care patients. Patients are admitted to a separated space or an individual room with a clock, visual and auditive contact with the staff, and the possibility to listen to the radio or watch television. Most of the patients have a window with visible daylight. All non-intubated patients with a score of at least 10 on the Glasgow Coma Scale, a minimum age of 18 years, and a stay of at least 24 hours before the first assessment in the ICU were included. Patients of all units were included, resulting in a mixed intensive care population in this study.

A trained nurse researcher included the patients once daily in the morning. First, the patient was assessed with the NEECHAM scale without calculating the results and immediately afterwards with the CAM-ICU. A test with the CAM-ICU was regarded as positive for delirium scoring positive on the algorithm. The NEECHAM scale categories were used to classify the patient. A test score of lower than 20 (moderate to severe confusion) is defined as 'delirium'. Each patient scoring positive for delirium at least once on the CAM-ICU or the NEECHAM scale was identified as delirious for the calculation of the incidence rates.

The included patients were classified in three categories of admittance: cardiac surgery, non-cardiac surgery, and internal medicine. Age, gender, and Simplified Therapeutic Intervention Scoring System 28 (TISS 28) score [14] were collected for all included patients. The mean TISS 28 score was calculated for each patient based on all daily values obtained during the stay in the ICU. The Acute Physiology And Chronic Health Evaluation (APACHE) II score is not validated for calculating the severity of disease or risk prediction for a cardiac surgery group. This score was calculated at the first day of admittance for the internal medicine and the non-cardiac surgery groups only.

To compare the studied scales, diagnostic descriptives were calculated in a two-by-two table for all paired assessments. Sensitivity, specificity, negative predictive value, and positive predictive value of the NEECHAM scale refer to the CAM-ICU as the reference assessment tool [15,16]. Subgroup analysis for age, gender, length of stay, and category of admittance was performed based on the most severe CAM-ICU and NEECHAM scale score of each patient.

The Statistical Package for the Social Sciences 14.0 (SPSS Inc., Chicago, IL, USA) was used for the statistical analysis. The different categories of admittance were compared using the chi-square test, the independent *t *test, and the one-way analysis of variance where applicable. Correlations were calculated using the Pearson correlation coefficient. Significance was calculated on a 0.05 level.

The protocol of this study was presented to the ethical board of the University Hospital of Antwerp, where it was approved. An informed consent was requested from the patient or his or her legal representative where appropriate.

## Results

A first group of patients was included in July to August 2006 and a second group in February to March 2007, resulting in a consecutive sample of 172 patients and a total of 599 paired observations. The mixed intensive care population was composed of 23% cardiac surgery, 37% non-cardiac surgery, and 40% internal medicine patients. The mean age of the included population was 60 years (range 20 to 90) and 59% were male. The mean APACHE II score was 21 (range 7 to 47) and the mean TISS 28 score was 29 (range 2 to 46) (Table 1).

**Table 1:** Description of the included population

	*n *= 172 patients	Cardiac surgery 23.3%	Non-cardiac surgery 37.2%	Internal medicine 40.5%	*P *value of difference
Age in years, mean (SD)	60 (14.9)	67 (10.2)	58 (14.4)	58 (16.4)	0.002^a^
Male gender	*n *= 102	28.4%	39.2%	32.4%	0.04^b^
Female gender	*n *= 70	15.7%	34.3%	50.0%	
APACHE II score, mean (SD)	20.6 (9.0)	-	20.1 (8.0)	21.1 (10.0)	0.65^a^
TISS 28 score, mean (SD)	28.6 (5.4)	32.7 (4.7)	28.4 (4.5)	26.5 (5.4)	<0.001^a^
Length of stay in days, mean (SD)	7.0 (8.9)	5.7 (8.5)	7.3 (10.2)	7.4 (7.9)	0.59^a^

^a^*P *value of difference calculated with one-way analysis of variance. ^b^*P *value of difference calculated with the chi-square test. APACHE, Acute Physiology And Chronic Health Evaluation; SD, standard deviation; TISS 28, Simplified Therapeutic Intervention Scoring System 28.

**Figure 1:**
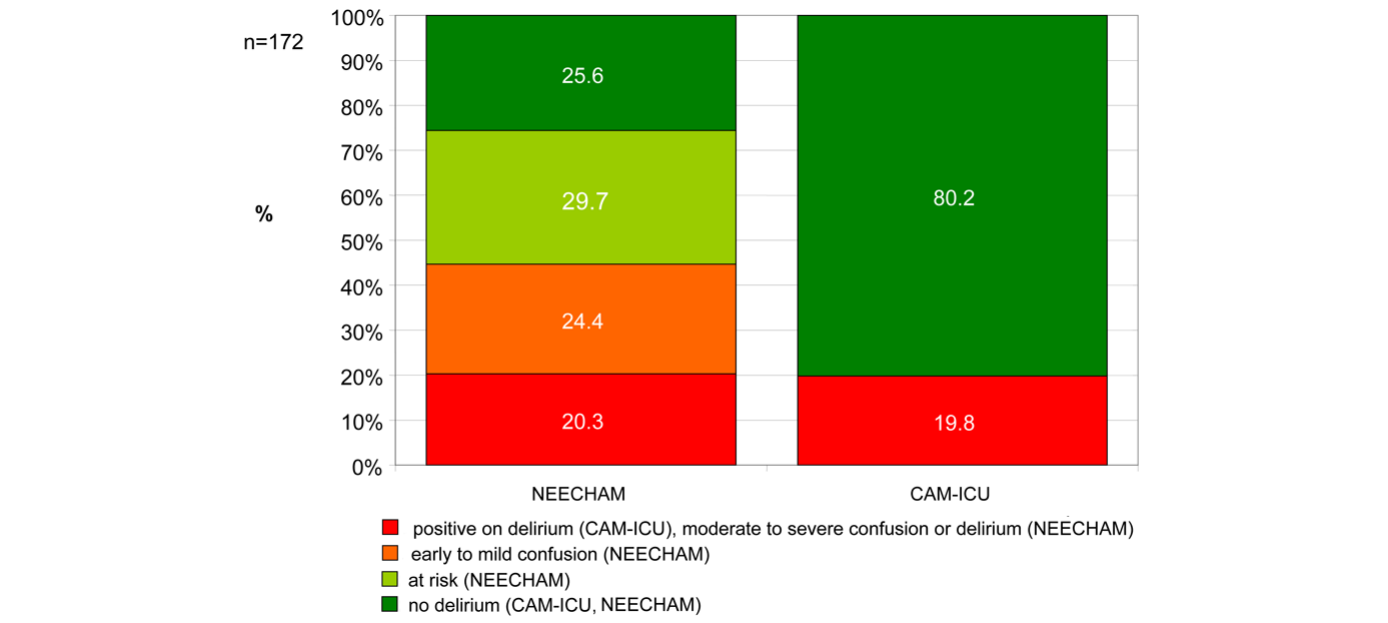
Incidence of intensive care delirium assessed with Confusion Assessment Method for the Intensive Care Unit (CAM-ICU) and Neelon and Champagne (NEECHAM) Confusion Scale (*n *= 172 patients).

The incidence of delirium assessed with the CAM-ICU was 19.8% for the total population. The NEECHAM assessment showed 20.3% with delirium, 24.4% with 'mild confusion', 29.7% as 'at risk', and 25.6% as 'normal' (Figure 1). Most of the patients scoring positive for delirium on the CAM-ICU were classified in the NEECHAM scale category diagnosing delirium. Almost a third of the patients scoring negative on the CAM-ICU were positive on the NEECHAM scale, most in the 'mild confusion' group and fewer in the delirious group. All of the patients scoring 'normal' or 'at risk' on the NEECHAM scale were assessed as negative on the CAM-ICU (Table 2).

**Table 2:** Distribution of the total population in a NEECHAM Confusion Scale versus CAM-ICU matrix

*n *= 172 patients	NEECHAM scale			
	
	Normal (n)	At risk (n)	Mild (n)	Delirium (n)
CAM-ICU normal, *n *= 138	44	51	38	5
CAM-ICU delirious, *n *= 34	0	0	4	30

'Mild' is defined as early to mild confusion. CAM-ICU, Confusion Assessment Method for the Intensive Care Unit; NEECHAM, Neelon and Champagne.

Positive delirium observations were obtained for 39 patients on 183 delirious days. Consequently, this resulted in a mean of 4.7 delirium days for each delirious patient, ranging from 1 to 18 days. Most of these patients suffered one (23%), two (18%), or three (13%) delirious days. Most of the delirious patients (31%) were positive for the first time within 3 days after admission to the ICU, and 57% were positive for the first time after 4 days. Within 7 days, 77% of the delirious patients were positive for the first time.

Subgroup analysis based on the most severe patient data (*n *= 172) showed similar results for the CAM-ICU and the NEECHAM scale. Both instruments agreed that there was no difference in the onset of delirium concerning age or gender (Table 3). Both showed a trend toward a higher incidence for the internal medicine patients. The length of stay in the ICU was higher for the delirious patients (Table 4). These results were significant regarding the CAM-ICU and the categories of the NEECHAM scale. Additionally, the NEECHAM scale scores showed a positive correlation with the length of stay in days (*r *= 0.61, *P *<0.01).

**Table 3:** Subgroup analysis for the incidence of delirium with CAM-ICU and NEECHAM Confusion Scale

*n *= 172 patients		CAM-ICU	*P *value	NEECHAM scale	*P *value
Age	Under 65 years, *n *= 98	22.4%	0.31	23.5%	0.24
	65 years or older, *n *= 74	16.2%		16.2%	
Gender	Male, *n *= 102	18.6%	0.65	19.6%	0.77
	Female, *n *= 70	21.4%		21.4%	
Category of admittance	Cardiac surgery, *n *= 40	15.0%	0.20	10.0%	0.08
	Other surgery, *n *= 64	15.6%		18.8%	
	Internal medicine, *n *= 68	26.5%		27.9%	

*P *value of the difference was calculated with the chi-square test. CAM-ICU, Confusion Assessment Method for the Intensive Care Unit; NEECHAM, Neelon and Champagne.

**Table 4:** Mean lengths of stay for delirious and non-delirious patients (CAM-ICU) and the four categories of the NEECHAM Confusion Scale

CAM-ICU	Mean length of stay in days (SD)	*P *value^a^	NEECHAM scale	Mean length of stay in days (SD)	*P *value^b^
Delirium	17.5 (14.5)	<0.001	Delirium	18.5 (15.1)	<0.001
			Mild confusion	7.0 (6.1)	
No delirium	5.0 (5.9)		At risk	4.0 (2.7)	
			Normal	2.8 (1.6)	

^a^*P *value was calculated with the independent *t *test. ^b^*P *value was calculated with one-way analysis of variance. CAM-ICU, Confusion Assessment Method for the Intensive Care Unit; NEECHAM, Neelon and Champagne; SD, standard deviation.

**Figure 2:**
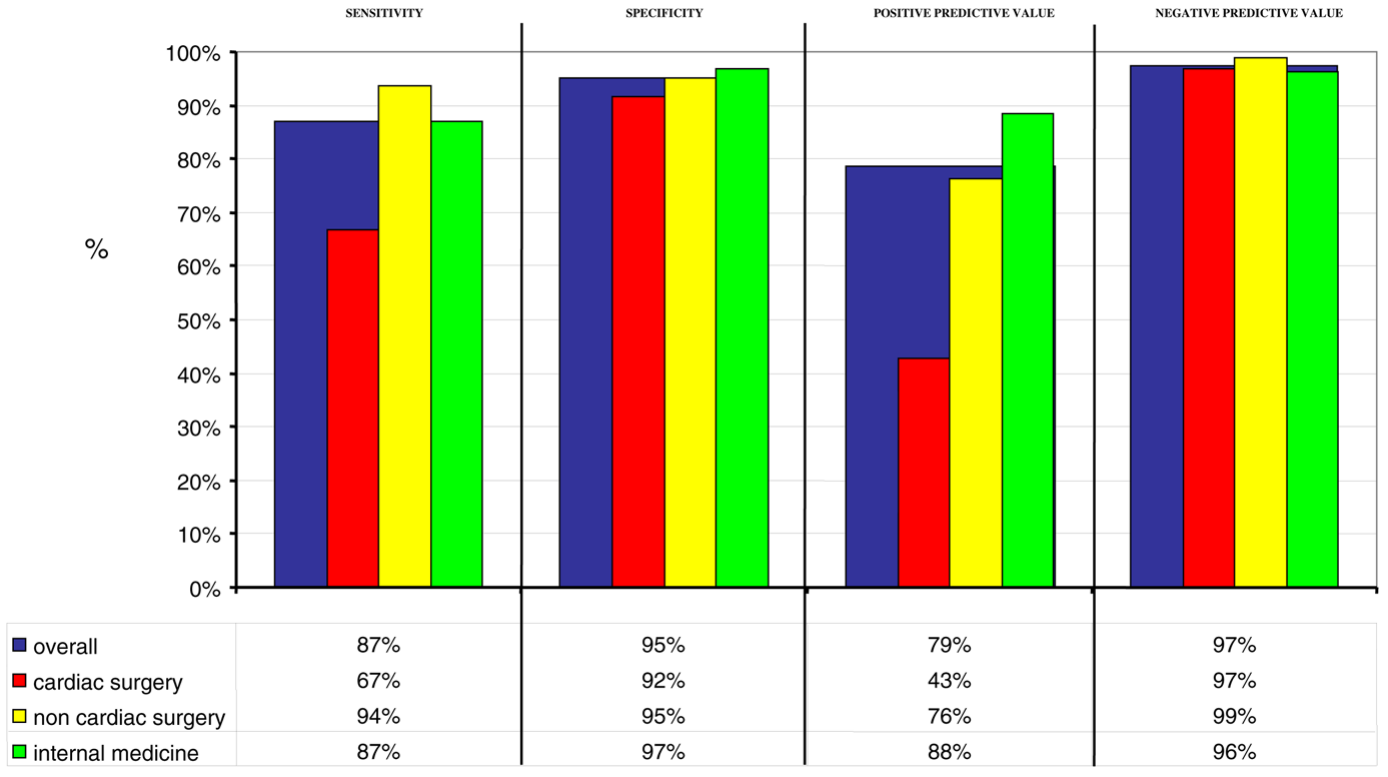
Diagnostic descriptives of the Neelon and Champagne (NEECHAM) Confusion Scale comparing to the Confusion Assessment Method for the Intensive Care Unit (CAM-ICU) as the reference tool. Values were calculated for *n *= 599 assessments.

Each NEECHAM observation was compared with the paired CAM-ICU observation to calculate the diagnostic descriptives (Figure 2). Using the NEECHAM cutoff value of less than 20 ('severe confusion'), test values were considered to be positive for delirium to calculate the diagnostic descriptives. The overall sensitivity was good but was lower in the cardiac surgery group (Figure 2). The specificity showed good results overall and in the different categories of admittance. Due to the lower sensitivity in the cardiac surgery group, the positive predictive value was poor for the assessment of this population but was higher in the other categories of admittance and was 79% overall. The negative predictive value was good overall and in the different categories of admittance.

## Discussion

In this study, the incidence of delirium assessed with the NEECHAM scale (20.3%) was comparable to the results of the CAM-ICU (19.8%). The diagnostic descriptives of the NEECHAM scale showed good results. Additionally, patients were classified in the different categories of the NEECHAM scale.

The research on intensive care delirium has taken a giant step forward since the development of assessment tools. A scale diagnosing delirium seems reliable when development was based on the DSM criteria. Hence, a confirmation by a psychiatrist is not necessary in daily practice. A gold standard for biological or physical tests, however, could be discussed [17]. A standard implies a level of perfection able to judge over all other tests. This perfection could hardly be attained by an individual assessing the patient.

Although the delirium assessment instruments have often been used in research, the implementation as a standard medical or nursing screening tool has just started in clinical practice. The CAM-ICU, the Intensive Care Delirium Checklist, and the NEECHAM scale are available to screen for delirium. Nowadays, there seems to be no need for the development of new tools, but the existing instruments should be studied thoroughly and refined to achieve a global understanding of the assessment of the delirium syndrome [18].

The CAM-ICU was developed for physicians and researchers based on the DSM criteria [19] but now is available to be used by intensive care nurses. The screening can be implemented in the daily nursing care after limited training. The instrument is translated and validated in 10 different languages. Therefore, the CAM-ICU usually is considered to be the 'gold standard' for the diagnosis of delirium. The incidence rates of delirium assessed with the CAM-ICU showed a wide range. Ely and colleagues [4,8] reported incidence rates of 83.3% and 87.0% in conscious medical or coronary care patients who were mechanically ventilated. McNicoll and colleagues [20] detected 31.1% delirium in medical intensive care patients older than 65 years, and Balas and colleagues [21] reported 28.3% in a surgical ICU. In our research, 19.8% of the mixed intensive care population developed delirium according to the CAM-ICU. The subgroup analysis of the internal medicine patients (Table 3) found an incidence of 26.5% in our population, but the other categories of patients developed less delirium. Our incidence rates assessed with the CAM-ICU seem to be lower than those of the published reports. This could be explained by the absence of ventilated patients in our population. Moreover, the architecture of the studied ICUs might play a beneficial role in the prevention of delirium (for example, the presence of visible daylight and a clock). Further research has to focus on the onset of delirium and the precipitating risk factors in the studied ICU.

The NEECHAM scale was developed as a nursing screening instrument for the early detection of delirium and was validated against DSM criteria for use in an ICU [13]. In this validation research, 19.4% delirium and 15.8% mild confusion rates were found in a medium-sized ICU of a general hospital. The population in our study had a similar incidence for delirium but a higher incidence for 'mild confusion'. A report of Csokasy and Pugh [12], also using the NEECHAM scale, showed a total score of 47% for both categories taken together. The patients in their population (*n *= 19) were all older than 65 years and were admitted to an ICU of a smaller hospital. As already stated by Immers and colleagues [13], the evaluation of the physiological condition may not be relevant to the delirium assessment of the patient in the ICU. Since there has been no research or validation study to verify this suggestion, the assessment of the physiological condition will be retained as a basic element of this tool. Additionally, further study is needed to adapt and validate the NEECHAM scale for the delirium assessment of the intubated or the ventilated patient. Also, a longitudinal study needs to inquire whether the numbered approach and the different categories of the NEECHAM scale have a predictive value against a binary approach. Consequently, the categories 'at risk' and 'mild confusion' could have an additional value. Preventive actions eventually could protect patients from becoming delirious. As Devlin and colleagues [22] in their excellent review of delirium instruments for the ICU already remarked, all evaluations are dichotomous and therefore do not measure delirium severity.

Besides the NEECHAM scale and the CAM-ICU, the Intensive Care Delirium Checklist is a commonly used screening tool for the detection of delirium in the ICU [23]. Incidence rates of 19.2% and 31.8% were reported in an adult population in a mixed ICU [24,25]. Many items in this scale can also be scored by a nurse during daily practice. This eight-item scale also provides a numeric approach to the delirium assessment. Each item scoring positive gets one point. A score of four points was considered to detect 99% of the delirious patients. A definition of a population 'at risk' or with 'mild confusion' is not provided. A binary approach of the score was suggested. Given the four categories of the NEECHAM scale, the last one creates more opportunities to classify the patient.

Four positive CAM-ICU patients scored 'mild confusion'. Five patients scoring negative on the CAM-ICU scored delirious on the NEECHAM scale. Four of them had a borderline score on the NEECHAM scale. One patient had a score of 14 on the NEECHAM scale and was assessed as negative for delirium on the CAM-ICU. This patient received propofol (through a continuous intravenous infusion pump), which possibly influenced the results. The NEECHAM scale proved to be a good delirium screening instrument with a strong denial power. The specificity proved to be good in all categories. The diagnostic descriptives for the NEECHAM scale in the cardiac surgery group, in contrast to the results of the other categories of admittance, were low.

Nurses are the first caregivers to observe the patient and to detect an altering cognitive function. The NEECHAM scale uses the daily observation skills of nurses and their standard 24-hour monitoring of a patient in the ICU. The CAM-ICU needs a short visual or auditive test. Both scales, showing the same result in the diagnosis of delirium, could be considered for implementation in the standard nursing observation or monitoring in the ICU. The focus in research on intensive care delirium should shift from possible treatments to early prevention of the syndrome [26,27]. The detection of patients in an early stage of confusion and the classification in categories could become an important advantage of the NEECHAM Confusion Scale [18,28]. Therefore, a longitudinal study is needed.

Our study is limited by the size of the population in the different categories of admittance. Each category could be the subject of a further study. Both studied scales were validated and verified for the intensive care setting. For the purpose of this study, a confirmation of the delirious state by a psychiatrist seemed unnecessary. The patient was assessed once in the morning. The simultaneous assessment of both scales could have created an interscale bias. The result of the NEECHAM scale, however, was calculated only after the paired assessment of the patient. Assessment of the patient at least three times a day could be recommended. A standardized screening for delirium should contain one observation during each nursing shift and an additional score on suspected events due to the fluctuating nature of the syndrome. The incidence in this study could have been higher when more daily assessments were completed. In addition, no ventilated or intubated patients were included. These categories of patients often develop delirium. There is a need to test the NEECHAM scale in this population.

## Conclusion

The scales showed a comparable incidence of intensive care delirium in our population: 19.8% for the CAM-ICU and 20.3% for the NEECHAM scale. Additionally, patients could be classified as 'early to mild confused', 'at risk', or 'normal' using the NEECHAM scale. The studied scale showed acceptable sensitivity, specificity, and predictive values. The cutoff value of 20 of the NEECHAM scale is valuable in the assessment of intensive care delirium. The scale uses existing nursing skills to assess the patient and is easy to implement as a screening tool in standard nursing observation.

## Key messages

• The Confusion Assessment Method for the Intensive Care Unit (CAM-ICU) and the Neelon and Champagne (NEECHAM) Confusion Scale showed comparable incidence rates of intensive care delirium: 19.8% and 20.3%, respectively. Additionally, patients could be classified as 'early to mild confused', 'at risk', or 'normal' by means of the NEECHAM scale.

• The NEECHAM scale showed acceptable sensitivity, specificity, and predictive values in comparison with the CAM-ICU.

• The cutoff value of 20 of the NEECHAM scale is valuable in the assessment of intensive care delirium.

## Abbreviations

APACHE = Acute Physiology And Chronic Health Evaluation; CAM = Confusion Assessment Method; CAM-ICU = Confusion Assessment Method for the Intensive Care Unit; DSM = *Diagnostic and Statistical Manual of Mental Disorders*; ICU = intensive care unit; NEECHAM = Neelon and Champagne (Confusion Scale); TISS 28 = Simplified Therapeutic Intervention Scoring System 28.

## Competing interests

The authors declare that they have no competing interests.

## Authors' contributions

BVR conceived the study, was responsible for the data collection, drafted the manuscript, and participated in discussing the results and revising the article. LB participated in designing and coordinating the study, discussing the results, and revising the article. ME assisted in the statistical analysis and participated in discussing the results and revising the article. MJS, ST, and LMS-B participated in discussing the results and revising the article. All authors read and approved the final manuscript.

## Appendix

Appendix 1

The Confusion Assessment Method for the Intensive Care Unit (CAM-ICU).

Appendix 2

The Neelon and Champagne (NEECHAM) Confusion Scale.
